# Changing the odds: motives for and barriers to reducing HCV-related sexual risk behaviour among HIV-infected MSM previously infected with HCV

**DOI:** 10.1186/s12879-018-3571-1

**Published:** 2018-12-18

**Authors:** Femke Lambers, Wendy van der Veldt, Maria Prins, Udi Davidovich, on behalf of the MOSAIC study

**Affiliations:** 1 Department of Infectious Diseases, Research and Prevention, Public Health Service of Amsterdam, Amsterdam, the Netherlands; 2 Department of Infectious Diseases (Centre for Infection and Immunology Amsterdam (CINIMA), Academic Medical Centre (University of Amsterdam), Amsterdam, the Netherlands

**Keywords:** HCV, HIV, Men who have sex with men, Sexual health, Protection motivation theory, Qualitative

## Abstract

**Background:**

Among HIV-infected MSM who have been treated for HCV infection, the HCV reinfection rate is high. It is therefore essential to understand their perceptions of HCV risk behaviour and risk-reducing strategies.

**Methods:**

This qualitative study among 20 HCV-infected MSM, the majority treated in the era before direct acting antivirals, provides insight into their ideas, motives, and barriers concerning HCV risk reduction, and aims to strengthen prevention strategies for both primary HCV infection and HCV reinfection.

**Results:**

The strongest motive to implement risk reduction strategies was the reward of avoiding HCV retreatment and its side effects, but this may change with the current implementation of less burdensome HCV treatment. Also, the sexual risk norms in the MSM scene, including social pressure towards risk-taking, HCV stigma, and non-disclosure of HCV status, all form barriers to safe sex. Drug use, strongly present in the context of clubs and group sex, directly impedes the self-efficacy of men to take risk reduction measures.

**Conclusions:**

Tailored prevention messages, empowerment of self-efficacy for risk reduction, and more insight into risk behaviour over time are ingredients for effective HCV prevention among these men.

## Background

The emergence of an epidemic of hepatitis C virus (HCV) infections among HIV**-**infected men who have sex with men (MSM) has become a new public health problem in high**-**income countries [1, 2]. Previously, the transmission of HCV, a bloodborne virus, was exclusively related to injecting drug use, surgical procedures, or usage of contaminated blood products. Studies among heterosexuals found the risk for sexual transmission of HCV to be extremely low [3]. Prevention of HCV infection was therefore focused on harm reduction strategies such as needle exchange programs, methadone provision, and screening of blood products [4]. However, the main risk factors for HCV transmission in HIV-infected MSM appear to be sexual [2]. Several studies have demonstrated that sexual practices such as fisting and sharing of toys, but also unprotected anal intercourse, specifically in a group**-**sex setting and under influence of drug use, are associated with acute HCV infection among HIV**-**infected MSM [5–7]. Unlike HIV infection, HCV infection can be cleared spontaneously or, in the last three decades, by increasingly successful treatment [8–10]. Until 2011, treatment of HCV consisted of weekly peg**-**interferon in combination with daily ribavirin, with modest success rates and side effects such as depression and anemia. Since the introduction of direct acting antivirals (DAA), it is highly successful with fewer side effects [11]. Accessibility to DAA differs according to disease stage (grade of fibrosis) and differs per country. Also, DAA is not yet approved for acute HCV infection in every country.

Most important, HCV clearance does not preclude reinfection if risk exposure continues. The incidence of HCV reinfection in the HIV**-**infected MSM population is high, at 5**–**15 per 100 person**-**years [12–15]. This suggests that some MSM are not able to implement HCV risk reduction measures after HCV clearance. To diminish the HCV epidemic among MSM, it is necessary to understand the bottlenecks for the implementation of risk reduction strategies. It is important to understand the motives regarding the practice of HCV**-**related sexual risk behaviour and the motives and barriers as to application of risk**-**reducing strategies. Such information will help strengthen strategies to prevent both primary HCV infection and HCV reinfection. The purpose of this qualitative study among 20 MSM with previous HCV infection was to provide this much**-**needed information.

## Methods

### Recruitment and sample

The study is based on in**-**depth interviews held from January 2011 to December 2012. In total, 19 HIV**-**infected MSM who were HCV**-**co infected, plus 1 HCV mono-infected MSM were interviewed to gain insight into their motives and barriers as to implementation of HCV risk reduction strategies. Men were recruited from the ‘MSM Observational Study of Acute Infection with hepatitis C’ (MOSAIC) at several HIV outpatient clinics or through the HCV facebook page of ‘Poz and Proud,’ a subcommittee of the HIV Organization Netherlands, a non-governmental organization which works in the interests of persons infected with HIV. Written information on the study was provided by the HIV nurse or study coordinator at recruitment. If men chose to enroll, they were invited by phone or email for an interview at their regular HIV**-**treatment location or at the offices of the Public Health Service of Amsterdam, depending on their preference. Recruitment continued until data saturation was reached, i.e. until no new motives or barriers to reducing sexual risk behavior and using risk reduction strategies emerged during the interviews.

### Procedure

In total, 20 face**-**to**-**face in**-**depth interviews, one per participant, were conducted (average duration: 60**–**75 min) by a medical anthropologist trained in qualitative research methods. At the beginning of each interview, the participants were provided with information on the aim of the study and the established conditions for anonymity and confidentiality, followed by an oral informed consent. The interviews were semi-structured and allowed the interviewer to deeply explore the participant’s feelings and perspectives on the relevant subjects.

The main focus concerned the impact of sexually**-**acquired HCV infection on sexual risk**-**taking and the motives and barriers to reducing risk behaviour. A topic list (Table 1) was used to initiate rapport between interviewer and participant, and themes were developed according to participant disclosures. The interviewer was free to pursue areas of interest or to probe responses further for clarification. All interviews were audio**-**taped and transcribed verbatim. Participants received a gift certificate (€20) for their participation.

**Table 1:** Main themes from topic list used during interviews with 20 (previously) HCV-infected MSM in the Amsterdam area from January 2011 to December 2012

Circumstances of HCV diagnosis	- Knowledge about HCV prior to diagnosis - Sexual behaviour prior to diagnosis - Feelings about HCV diagnosis
HCV infection	- Current HCV status - Perception of HCV since diagnosis - Experiences with treatment of HCV - Knowledge about reinfection
Sexual behaviour	- Current sexual behaviour - Sexual behaviour between diagnosis and treatment - Sexual behaviour during HCV treatment - Sexual behaviour after HCV treatment
Risk reduction strategies	- Risk reduction strategies prior to HCV infection - Risk reduction strategies after HCV infection - Influences of HCV infection on risk reduction - Perception of HCV risk in future - Perception of self-efficacy of risk reduction

### Theoretical background

The Protection Motivation Theory was used as a theoretical base for the interpretation of the data. Protection motivation refers to the motivation to protect oneself against a health threat like an HCV infection. The theory proposes that the intention to protect oneself depends upon two concepts. One consists of the *threat appraisal* of a disease, in this case HCV infection: its perceived *severity*, one’s perceived *vulnerability* (probability of becoming infected), and the perceived *rewards* of implementing preventative measures. The other concept consists of the *coping mechanisms* for implementing protective measures: the perceived *response efficacy* of the measures (the belief that they are truly preventative), the perceived *self-efficacy* (the belief that one is able and likely to perform the preventative measures), and the *costs* of their implementation [16]. The probability of implementing HCV risk reduction strategies increases with higher perceptions of HCV severity, one’s vulnerability to HCV reinfection, response efficacy, self-efficacy for implementing the strategies, and rewards of preventative behaviour. When one or more of these perceptions/factors is low, behavioural change to protect against HCV reinfection is less likely to occur.

### Analysis

The transcribed interviews were entered into a database for coding, using qualitative data analysis software (MAXQDA 10) which allowed for coding of data segments using a flexible set of categories. In the first phase of data analyses, open coding by two researchers (a medical doctor and a medical anthropologist) was performed to explore the subjective experiences of the participants regarding HCV diagnosis, treatment, sexual behaviour, risk reduction, etc. Transcripts were repeatedly examined to discover emerging themes which were discussed and, if found relevant, added to the initial topic list of the interviews. This iterative method allowed us to adjust data collection according to what was learned [17] and ultimately to establish saturation. The two researchers coded the same data from all transcripts to ensure that codes being used were the best fit for the segments. Where disagreements were noted, they discussed their difficulties, clarified their opinions, and agreed to a final classification after discussion with the study supervisor, a social and clinical psychologist. Upon reaching consensus, the researchers continued to perform coding to excerpt and sort segments of data into subcodes. In the second phase of analyses, focussing on the research aim, the researchers grouped relevant codes into categories based on the theoretical background of the Protection Motivation theory. They produced a list of categories covering motives and barriers to changes in sexual behaviour for use in analyzing the remaining transcripts. This procedure was followed until a definite database was created containing a definite list of categories. The final results, interpretations, and conclusions were based on that database and supported by quotes from the interviews.

### Ethical framework

This study is part of the MOSAIC study which is approved for by the Medical Ethics committee of the Academid Medical Center in Amsterdam, the Netherlands.

Information on the aims, procedures, and use of findings was provided to each participant. Participants were informed that answering the interview questions was voluntary and they could withdraw from participation at any time. During the consent process, participants were informed about the confidentiality of transcripts and publications and assured that all identifying markers would be removed. The consent was recorded on audiotape and documented by transcription together with the interview.

## Results

### Characteristics

Participants’ average age was 39 years (range 27**–**59), most were born in the Netherlands (95%), and approximately half of the men were in a steady relationship. Of the 20 participants, 5 were still undergoing HCV treatment during the time of the interview; 11 had been successfully treated in the (recent) past; 2 had been unsuccessfully treated and were chronically infected; 1 was chronically infected and had never received treatment, and 1 had spontaneously cleared the HCV virus. Of the 5 in treatment, 4 men had contracted HCV more than one time: 2 had been reinfected once, and 2 had been reinfected for the second time.

### Interview results

First, we will describe the risk reduction strategies that were implemented by the participants, followed by the motives and barriers to changing sexual risk behaviour and applying HCV risk reduction strategies.

### Implemented HCV risk reduction strategies

Participants described a wide range of strategies used to prevent HCV reinfection.

#### Preventing direct transmission

Several strategies concern reducing direct transmission of HCV during sex, e.g. using condoms; not sharing gloves, toys or lubricant; and limiting mucosal damage:

“No sharing of gloves, condoms, toys, et cetera (….), surely these kind of essential things, which may give blood-to-blood contact (.….). Well, using a toy or something like that, while being with more persons at the same time, it could happen that we exchanged it. But now [after HCV diagnosis] I’m very cautious that this won’t happen to me again.” (MSM 5).

“Sometimes Crisco [lubricant] is being used. So then you should be using your own jar. If you go through it [Crisco] and then someone else goes through it, you could just as well have unsafe sex.” (MSM 4).

“Using drugs anally is something I definitely don’t do anymore (…). I suspect it is unhealthy for the mucosa, by which you increase the risk of being more susceptible to not only HCV but also other STI.” (MSM 8).

#### HCV serosorting

Some strategies to diminish risk of reinfection depend on choosing sex partners based on their assumed HCV status:

“When someone is HCV-infected, I don’t want sex with him. Just because of the risk of infection.” (MSM11).

“Well, we still have unsafe sex with each other, but not at the moment, because he is HCV-infected and I absolutely don’t want to become infected again.” (MSM5).

#### Avoiding risk-related contexts

Some risk reduction strategies involve simply trying to avoid specific sexual contexts that are perceived by participants as encouraging risk. Group sex, sex in clubs, or sex under influence of drugs are perceived as contexts associated with risk**-**taking:

“During group sex at a club, you are more likely to meet somebody with HCV, because you have sex with more men. Also it is more likely to skip the use of a condom, especially near the end of the evening after four glasses of vodka. So I am eliminating that kind of group sex at those clubs. I am going one-on-one. That lowers the risk of contracting HCV.” (MSM19).

“I think drugs are a risk factor anyway, and that is why I would like to eliminate them.” (MSM11).

“I’m totally done with drugs. You know, I can say that drugs were partly the cause, and that’s true, but in the end I myself am responsible for my actions. So I did it myself. So I don’t want to blame it all on drugs, but drugs have certainly been a catalyst. If I hadn’t used it, I would have certainly used a condom during sex.” (MSM17).

#### Being cautious on the internet

Negotiating safe sex with partners met through internet dating is perceived to be challenging, as disclosure of HIV and HCV status and safe**-**sex intention are sometimes not apparent or transparent over the internet. Some men are therefore more cautious when dating through the internet or using the information put online to screen their choices:

“I will never date somebody who fills in ‘needs discussion’ [answer to question about safe-sex intention on internet profile], because that means that they do not always have safe sex. And I can’t trust them, especially when drugs are involved.” (MSM18).

*“Do you make new contacts through the internet?” (interviewer).*

“We really don’t feel like that anymore. We think that world can’t be trusted. They never say what’s going on, what they have. That risk is too big for us. You never know what you get into your home. No, we don’t do that anymore.” (MSM2).

### Motives for implementing risk reduction strategies

#### Effect of diagnosis and treatment on sexual desire and self-perceived attractiveness

Men reported changing behaviour shortly after start of HCV treatment due to its impact on sexual desire. Many men suffered from side effects such as extreme fatigue, flulike symptoms, and depression which lowered sexual desire and the ability to take sexual initiatives. Reducing sexual behaviour during treatment could therefore be not so much driven by deliberate rational or emotional motives but rather by physical circumstances:

“The medication is heavy, I agree; because of that you don’t have sex anymore, you don’t feel like going out, you don’t do any drugs. So, the medication is heavy, but the choices you have to make are actually made for you, which is very easy. But what happens when you have finished the treatment?” (MSM 5).

“You get a lot of side effects, you feel really sick, every weekend again, so you won’t be able to do a thing.” (MSM15).

Moreover, men feel too unattractive to have sex, partly due to the stigma of HCV infection:

*“Did the HCV diagnosis influence your sexual life? You mentioned that at the beginning you did not feel like it [having sex].” (interviewer).*

*“*Well, I felt dirty. You know, dirty not in a sense that nobody was allowed to touch me but, well, there is quite a taboo against HCV. It is also being called the new HIV, comparable to some years ago. HIV is sort of seen as something normal, but HCV is not.” (MSM 6).

#### Fear of HCV treatment side effects

Almost all participants perceive HCV to be a severe infection, like or even more severe than HIV infection or other STI. This is mainly due to the negative perception of HCV treatment. As stated above, HCV treatment side effects have a strong impact on physical and mental health and day**-**to**-**day life, including work, social life, and relationships, and are therefore mentioned as an important motive for behavioural change:

“Well, you see, I always say I would a hundred times rather have an HIV infection than an HCV- infection, and I really mean it. Because HIV really means nothing compared to it [HCV].” (MSM9).

“That’s what [HCV] changed my behaviour. You see, an infection with gonorrhea or chlamydia sometimes means taking a pill, not a treatment you want very often, but it’s not a disaster when it sometimes happens. But this [HCV] is really something that has great impact and simply takes six or, when unlucky, twelve months of your life. It’s not worth it.” (MSM5).

“It is such a demanding treatment, if I were to go through it again, I fear things might go wrong. I simply don’t want that to happen. So from now on I do it safe or not at all.” (MSM3).

#### Fear of infecting others

In addition, an often mentioned motive for changing behaviour is the fear of infecting somebody else:

“both times that I was HCV-infected I did not have sex during the treatment period...[tells reasons]. I also wanted to be sure that I did not infect anybody with HCV.” (MSM19).

#### Knowledge and high perceived susceptibility

The fear of becoming infected yourself is related to the perceived personal susceptibility for HCV infection. For some, experiencing previous infection and subsequently gaining knowledge about transmission has increased the perceived susceptibility for a new infection and contributed to changing behaviour in the future:

“Well, on the other side, I wasn’t totally aware how easy you can become infected with HCV. I always assumed you really had to do a lot: so really have rough sex or long-lasting sex, you know, that there really should be blood. That’s what I thought, about the way you get HCV through sex, and that’s not what I did. Now that I know that the HCV virus can stay alive outside your body for a long time, it becomes a whole different story […] If I had known that back then, I would not have done that.” (MSM11).

### Barriers to implementing HCV risk reduction strategies

Although most participants were motivated to implement one or more of the above**-**mentioned risk reduction strategies, a wide range of barriers to do so was mentioned.

#### Lack of HCV knowledge and low response efficacy

The intention of implementing risk reduction strategies may be absent or hampered when the participant does not know how HCV transmission can be prevented or does not believe in the efficacy of preventative strategies:

“Well of course you have this idea, you can contract anything, when you don’t know how you contract it, you can’t prevent it anyway. I might better live in a bubble then. I mean, you can’t protect yourself if you don’t know what to protect yourself against. It’s no use.” (MSM7).

“I should stop having any sex at all, because it really doesn’t matter what I do to prevent HCV. Whether I am having safe sex or not, I will contract it either way.” (MSM19).

#### Heat of the moment

Sex has been described as a non**-**rational act that does not always line up with considering and actively introducing protective measures in the heat of the moment:

“It’s like, postponing thinking about the possible consequences at the moment you have sex; the heat of the moment, but also thinking that you won’t catch it again. Afterwards, you naturally think ‘how stupid of me’.” (MSM19).

#### Negative impact on sex quality

The use of condoms can negatively influence an erection, and is therefore an obstacle to performing sex:

“With a condom, it’s always difficult and because it’s a hassle, you lose your erection. It makes me nervous because I’m not used to it.” (MSM 11).

#### Challenging settings

Certain sexual contexts, such as club settings or group sex are described as having an impact on the capability to use condoms or implement other risk reduction strategies:

“Sometimes I go to certain clubs. It’s much easier there to let go of certain things and to be less careful than you normally are [….] It’s the group process and also exhibitionism, to a certain extent.” (MSM19).

“[...] sometimes such a party goes on for two days and at a certain point people just go on, and things get a bit, well, messy: a hand goes in or a dildo, I don’t know. Thinking becomes less sharp.” (MSM17).

“No, you’re not with the same partner the whole evening. It happens, but mostly not. No, four, five, six....At a certain point, hands don’ t get washed that well anymore.” (MSM12).

#### Drug use

Drug use was reported to lead to impulsivity and to impede the ability to consistently implement risk reduction strategies.

*“During which situations did it [safe sex] go by the board?” (interviewer).*

“When you’re under influence of drugs, I would say. You become less careful, you’re not really aware anymore.” (MSM2).

#### Sexual risk habits and peer pressure

A commonly mentioned barrier is the habit of not using condoms when those around you are not using them either. Many men became part of the “bare**-**scene” after having been diagnosed with HIV. They became used to not needing a condom anymore to prevent HIV infection. Breaking that norm by re-starting condom use to prevent HCV**-**infection proves to be difficult:

“In a very short period, the behaviour of many people changed [to bare sex], including mine.”

*Do you have an explanation for that?*

“Yes, I do. Firstly I think that at a certain moment it’s almost strange to use a condom if everybody does it [bare sex]. That’s because the risks and the quality of [HIV] treatment have improved. It’s kind of backwards: just because you know that your life is not (or less) at stake, you start taking more risks.*”* (MSM5).

“Every person that’s [HIV] positive, in my surrounding, does it unsafe.”

*“So, unsafe sex is actually the norm?”*

“Yes, for positive persons it is.” (MSM6).

“It’s not like just using a condom again, it’s a total switch in your behaviour.” (MSM14).

#### Disclosure-related barriers

Being part of a sex environment that does not use condoms also leads to the fear of losing sexual partners when making HCV infection and condom**-**use part of sexual negotiation, as this necessitates the disclosure of the HCV status. Participants confirmed that disclosure of HCV status is therefore complicated and often not done:

“It’s not desirable to contract HCV, certainly not since most are already HIV-positive. They feel like, ‘That’s something I can’t handle [on top of HIV] as well, and I won’t tell it once I have it.’ It’s like that for me, because I don’t tell it either. I feel like, no one is talking about it, why should I? If I would, I know for sure that I won’t receive any invitations [for sexual contact] anymore.” (MSM17).

In addition, for men who have not disclosed their HIV status, the disclosure of HCV status can mean two disclosures at once:

“When someone hears hepatitis C and starts googling it, then it becomes evident that the person in question is also HIV-positive […] That feels like a nasty blow. I thought, I’m doing it the wrong way, I should not disclose it this easily.” (MSM18).

Open disclosure of HIV or HCV status online occurs often. The internet is a low-threshold way to meet sexual partners and is often used by MSM. Statements about wanting to have safe sex only or about one’s negative HIV/HCV status are sometimes visible on online user profiles. However, study participants have experienced that these statements are not always trustworthy:

“It’s not like, when somebody says safe sex [preferred type of sex expressed on the internet profile], it will automatically be like that.” (MSM5).

“Well, then [when posting you want condomless sex] you actually show you are HIV- seropositive. Consequently you also sort of admit you are HCV-positive. So, you actually contradict your own status. Many rather not do that. So, you post ‘safe’.” (MSM17).

“On the other side, I have also had a chat session with somebody literally asking me ‘you don’t have an STI?’ My thoughts at that moment were: I’m not going to tell you I have HCV, I don’t even know you. I mean, you will pull out, but what will you do with that information? You’ve got my chat name, perhaps we have contacts in common, so it’s easy to blab to others about it.”

*“So what did you do?*” (interviewer).

“I told him I did not have anything.” (MSM18).

*“And you met up with him?*” (interviewer).

“Yes.” (MSM18).

*“And the subject was not brought up again?” (interviewer).*

*“*No.” (MSM18).

“(…..) *So, it’s difficult to make it open to discussion?” (interviewer).*

*“*Yes, it’s really very complicated.” (MSM18).

#### Mental health

Some men reported their ability to implement risk reduction was influenced by their mental condition. Symptoms of depression are described as posing another barrier for implementing risk reduction strategies:

“(…) when you care less about yourself, (…), a sort of neglect [takes place]. That’s in fact what in a way also happened to having safe sex.” (MSM 16).

#### Declining perceived severity due to decline of side effects

When treatment has been successful, the motivation to prevent HCV reinfection may decline due to the fact that the experienced discomfort declines:

“I mean, the first months [after HCV treatment] you start feeling better and you have good intentions; it’s not going to happen to me again. But I know what I’m like. At a certain moment, when it’s a year later, you start crossing your boundaries again, doing this and that.” (MSM5).

## Discussion

The main focus of this study concerns the impact of sexually acquired HCV infection on sexual risk behaviour among HCV**-**infected MSM, exploring which risk reduction strategies are being implemented and what are the motives for and barriers to their implementation.

We will discuss our results within the theoretical framework of the Protection Motivation Theory and existing literature. Accordingly, we will suggest strategies to improve interventions for MSM with and at risk of HCV (re**-)**infection.

### Threat appraisal of HCV infection

#### Severity

In contrast to another qualitative study [18], possible complications of HCV infection such as chronic liver disease and its maligne interaction with HIV infection did not profoundly contribute to the perceived threat of HCV among our participants. It could be that the fear of complications was not specifically mentioned during the interviews because other topics, such as the severity of HCV treatment, were more salient. It could be that expectations of favourable treatment outcome are predominantly high. For treatment with peg**-**interferon and ribavirin, starting treatment shortly after diagnosis of acute HCV, the success rate of HCV treatment is indeed high, even in HIV**-**coinfected patients [19]. As a result, men perhaps do not perceive the severe consequences of untreated HCV infection as a likely scenario for their future, considering their high intention to get treated. Still, when treatment fails, complications such as liver cirrhosis can become severe and may occur more quickly as suggested by Fierer et al. [20]. Finally, our participants may simply be unaware of HCV complications, as was found among MSM participating in the Amsterdam Cohort Studies [21].

Addressing complications of HCV infection in the at**-**risk population through HCV**-**specific information campaigns can contribute to more accurate threat appraisal. Particularly when information is provided by HCV**-**infected peers, the consequences of infection may become more explicit and recognizable.

As the success rates of treatment are increasing with the introduction of new anti**-**HCV agents, physicians should clearly discuss with their patient the still possible side effects, the risk of reinfection and how to address it and, if relevant, the effort and financial cost that are involved.

#### Vulnerability

Besides the perceived severity of a disease, the perceived vulnerability to that disease determines the threat appraisal and accordingly the behavioural response.

Some of our participants did not perceive themselves as belonging to a high**-**risk group since they did not engage behaviour that they considered risky, such as group sex or techniques such as fisting. The emphasis of study results and prevention messages on the extremes of risky sexual behaviour as a main cause of infection seems to have anchored the feeling that *only* such behaviours can lead to HCV**-**infection. For some men, the fact that their infection occurred despite the avoidance of such extremes caused them to doubt the efficacy of certain risk reduction strategies. Consequently, while some men became more careful and started implementing more rigorous risk reduction strategies, some felt there are no realistic behavioural steps they can take to make themselves any less vulnerable. Ambivalent perceptions of risk make it challenging to develop effective prevention messages. Focussing on the most risky subpopulations and highest**-**risk techniques may help those most at risk, but it might also create the illusion that lower**-**risk techniques are completely safe from HCV infection. Tailored prevention messages need to be constantly updated as the transmission dynamics within a risk population change, e.g. when HCV spreads to HIV**-**negative MSM, presumably because of the protective effect PrEP (pre-exposure prophylaxis) has on the acquisition of HIV [22, 23]. Furthermore, addressing only high**-**risk subgroups may increase the stigma around HCV and indirectly raise a barrier to risk reduction strategies, as it increases avoidance behaviour and prevents free communication and HCV status disclosure [24]. Regular consultation on preventative interventions should offer realistic messages regarding high**-**risk techniques and should focus on the full range of techniques that can lead to HCV infection. Discussing such techniques should be paired directly with realistic suggestions of risk reduction strategies even for techniques that are considered more low**-**risk.

#### Rewards of applying risk reduction strategies

Classic HCV treatment (peg**-**interferon and ribavirin) often had a temporary but severely negative effect on patients’ well-being through serious physical and psychological side effects, and it consequently negatively influenced work participation, daily activities, sexual experiences and partnerships. Not wanting to go through these negative treatment**-**related experiences was an important motivator for behavioural change among our participants and was seen by some as an important reward for applying risk reduction strategies. Eventually though, treatment**-**related complaints will decline due to new less burdensome treatment options, with possibly a decline in motivation to prevent reinfection and an increase in risk**-**taking. Interestingly, this concern was expressed by participants themselves [data not shown]. Some expected that with new HCV treatment regimens, HCV infection will be ‘just another’ STI that is ‘easily’ treatable. Future studies should examine whether avoiding HCV retreatment is still a motive for risk reduction in the DAA-era, and close attention needs to be paid in future HCV prevention programs to the effect of new HCV treatments on declining motivation to change behaviour to avoid HCV reinfection. Counter measures could include communication of treatment costs and the low but existing possibility of treatment failure.

An additional motive to implement risk reduction strategies was the reward of not infecting somebody else. The issue of responsibility for one’s own or one’s partner’s health seems affected on the one side by social commitment and, on the other side, by fear of stigmatization after status disclosure. How these conflicting forces are resolved seem to determine whether a person will or will not engage in risk reduction behaviour or status disclosure with a partner. The balance between these motives should be addressed in prevention messages for both sides of the spectrum, both to increase responsibility towards sex partners and also to decrease HCV**-**related stigma.

### Coping appraisal of HCV risk reduction strategies

For many MSM, the barriers to risk reduction implementation are related to limited coping mechanisms for behavioural change. They question or feel challenged by both the response**-**efficacy of risk reduction strategies and the self**-**efficacy of applying these strategies.

#### Response efficacy

To start with the response efficacy, the ongoing discussion about risk factors for HCV infection in this population apparently leaves men in doubt about what risk reduction strategies are actually effective. Some men choose to be very careful and apply all recommended strategies; others find that they still might become (re) infected even when applying these strategies. The indistinctness of risk factors leads to lack of faith in the suggested strategies and results in a perception of low response efficacy. Providing a constant, clear, and uncomplicated prevention message linked to an accessible and tailored HCV testing policy may reduce doubts about response efficacy. Using an HCV risk score to determine the need for testing, as developed by Newsum et al. [25], may be such a method.

#### Self-efficacy

Although low response efficacy is a major issue for some men, the majority of our participants experience barriers related to low self-efficacy. Implementing risk reduction strategies such as condom use, proper glove usage, sterilization of toys, and separate and hygienic lubricant containers is challenging in certain sexual contexts such as group sex or public venues. Helping men to improve their skills for implementing these strategies and facilitating these strategies under these conditions can improve uptake.

External factors that influence self-efficacy are difficult to tackle. In the interviews it became apparent that interpersonal interaction and situational circumstances during sex have a high impact on the self**-**efficacy of implementing risk reduction strategies. Since the HCV epidemic has specifically affected HIV-infected MSM, a number of our participants confirmed being part of a social network of HIV**-**infected MSM who practice condomless sex. They stressed that being part of this scene creates peer**-**based and contextual barriers to HCV risk reduction strategies that are hard to overcome. The norms and social pressure during, for example, sex parties or group sex make it more difficult to disclose HCV**-**status and to request safe sex. The significance of condomless sex is multifold, ranging from pleasure**-**seeking to identity**-**forming [26, 27]. To change the norms within this social**-**sexual structure is challenging, but reducing HCV stigma and increasing awareness for HCV prevention within such networks is imperative. Innovative social-network approaches, e.g. using online social media and mobile phone technologies, can facilitate the dissemination of information and support within social and sexual circles and help make HCV a more overt topic of consideration.

In addition, lowering HCV testing thresholds and increasing test frequency could support men in high**-**risk sexual networks. For example, the development and dissemination of client**-**initiated HCV home**-**collection tests would facilitate frequent testing and distribution of tests among the high**-**risk networks.

Another important factor influencing self**-**efficacy among our participants was drug use. The percentage of HCV**-**infected MSM using recreational drugs before and/or during sex (“chemsex”) is high [7, 28]: 84% of MSM with acute HCV who participated in the MOSAIC study compared to 53% of MSM without acute HCV. The association of HCV infection with drug use may reflect a direct transmission route of HCV through sharing needles or snorting equipment [6, 7]. However, the negative effect of drugs on self**-**efficacy and thereby its association with other high**-**risk sexual behaviour is most probable. The specific drugs used, particularly ketamine and methamphetamine, increase sexual desire and endurance, but at the same time they lessen the capability for risk assessment and behavioral adaptation. This effect was strongly emphasized by our participants. It is one of the risk behaviours that men intend to change after HCV diagnosis, but being the norm in some sexual networks, drug use is difficult to reduce. For many, it has become the only way to perform and sustain their desired sexual activity, despite the danger of slipping into generalized addictive behaviour. Changing sexually**-**related drug use is challenging, and emphasizing its negative consequences is not sufficient to bring long**-**lasting change. HCV prevention measures could perhaps integrate components of drug use interventions, such as cognitive behavioural therapy or related interventions [29, 30], into their approach. Such interventions should be tailored to the personal context and possible comorbid mental illness of the user, while also building on existing interventions [31].

The challenges that come with condom use were mentioned by our participants as a behavioural barrier to risk reduction strategies. Condom use can negatively influence their erection and impede the intimacy experienced between partners [32]. This makes them less able to use condoms, even when they intend to. However, strategies effective for increasing condom use to prevent HIV and other STI may also be effective in preventing HCV. Motivational interviewing and skills training, combined with tailored cognitive behavioral interventions, are several of these evidence**-**based strategies [33].

Last but not least, as described by our participants, mental health problems may also reduce self**-**efficacy and can be exacerbated by HCV treatment with interferon. While depressive symptoms can reduce sexual desire, they can also make one indifferent to the threat of sexually transmitted diseases. The fact that syndemic factors such as mental disorders and drug use often coincide with high**-**risk behavior among MSM [34, 35] is an issue that public health workers and physicians should bear in mind when discussing safe sex and related topics. Even though patient with depressive symptoms receive professional attention during HCV treatment, more post**-**treatment attention is perhaps needed and would help to prevent HCV reinfection.

#### Costs of applying risk reduction strategies

Some men consider the costs of changing their sexual way of life (using condoms, reducing number of sex acts, giving up group sex) as too high. For them the pleasure of sex outweighs the risk of reinfection.

Furthermore, our participants indicated that reducing risk behaviour to protect others might directly or indirectly signal their HCV and HIV positivity to old or new sexual partners, leading to rejection. This is considered one of the highest costs of implementing risk reduction strategies within the existing sexual context of our study participants. A study among MSM by Owen et al., one of two other qualitative studies on HCV stigmatization*,* describes its negative consequences on status disclosure and its effect on implementation of risk reduction strategies [36]. Clearly, addressing issues of HCV stigma, generating more open discourse regarding HCV transmission, and improving HCV testing and cure in high-risk MSM are more imperative than ever. Empowerment of men through interactive online training modules that allow them to practice management of disclosure issues may be one method to facilitate this.

## Conclusions

With this qualitative study, we are among the first to describe the implementation of risk reduction strategies after HCV diagnosis among MSM previously infected with HCV, with emphasis on the motives and barriers to engage in such strategies. An important motive to implement risk reduction strategies was the reward of not having to go through peg**-**interferon/ribavirin treatment and its side effects again. This motive may lose strength in the future, as less burdensome treatments with DAA are becoming common practice. Future HCV prevention strategies should take such treatment**-**related changes in motivation into account. Instead of a focus on avoiding treatment, perhaps a more useful focus would be self**-**efficacy, since the lack of it was found to be a prominent barrier to behavioural change. Particularly sexual norms prevalent within the sexual network of HCV**-** positive MSM are described as difficult to manage and result in social pressure, HCV stigma, and non-disclosure of status. Drug use, strongly present in the sexual contexts of many HCV**-**infected MSM, also directly influences self**-**efficacy. Specific training modules targeting drug use in sexual contexts should be developed, evaluated, and integrated into HCV prevention initiatives. Prevention messages should be provided on a tailored basis, considering the dynamic nature of risk behaviour and sexual networks and therefore developed and disseminated with peer cooperation, possibly using online technologies and social networking. Each HCV**-**positive MSM’s thoughts and feelings about sexual activity, risk reduction, and HCV reinfection should be periodically explored after both HCV diagnosis and treatment to raise physician and patient awareness of possible changes in threat appraisal.

## Abbreviations

DAA: Direct Acting Antivirals
HCV: Hepatitis C Virus
HIV: Human Immunodeficiency Virus
MOSAIC: MSM Observational Study of Acute Infection with hepatitis C
MSM: Men who have Sex with Men
PrEP: Pre-Exposure Prophylaxis
STI: Sexually Transmitted Infection
